# Diet quality and therapeutic targets in patients with type 2 diabetes: evaluation of concordance between dietary indexes

**DOI:** 10.1186/s12937-017-0296-8

**Published:** 2017-11-21

**Authors:** Juliana Peçanha Antonio, Vanessa Costa da Rosa, Roberta Aguiar Sarmento, Jussara Carnevale de Almeida

**Affiliations:** 1 Endocrinology Division, Hospital de Clínicas de Porto Alegre (HCPA), Rua Ramiro Barcelos, 2350, Prédio 12, 4° andar, Porto Alegre, RS 90035-003 Brazil; 2 Universidade Federal do Rio Grande do Sul (UFRGS), Porto Alegre, Rio Grande do Sul Brazil; 3 Centro de Estudos em Alimentação e Nutrição (CESAN), HCPA/UFRGS, Porto Alegre, Rio Grande do Sul Brazil

**Keywords:** Dietary indexes, Diet quality, Diabetes mellitus type 2

## Abstract

**Background:**

This study aimed to evaluate the concordance between two dietary indexes, the Healthy Eating Index (HEI) and the Diabetes Healthy Eating Index (DHEI), in evaluating diet quality and its possible association with therapeutic targets in patients with type 2 diabetes.

**Methods:**

Cross-sectional study of outpatients with type 2 diabetes mellitus treated at a university hospital. Dietary information was obtained from a quantitative food frequency questionnaire (previously validated for use in patients with type 2 diabetes) and converted into daily intakes. Diet quality was assessed using two dietary indexes: HEI (12 components, nine food groups and three moderation components) and DHEI (10 components, six food groups, three nutrient groups, and one for variety of diet). In both indexes, the sum of the scores for each component yields an overall score converted on a scale from 0 to 100%; diet quality is subsequently ranked as low (<51%), needing improvement (51–80%), or high (>80%). Patients underwent clinical and laboratory assessment. Those with fasting blood glucose values 70–130 mg/dL, A1c < 7%, total cholesterol <200 mg/dL, LDL-cholesterol <100 mg/dL, and triglycerides <150 mg/dL were considered to meet therapeutic targets. All analyses were conducted in PASW Statistics 18.0, and *p* < 0.05 deemed significant.

**Results:**

We analyzed 148 patients with type 2 diabetes (73% white, mean age 63.2 ± 9.4 years, median diabetes duration 10 [IQR 5–19] years, mean A1c% 8.4 ± 2.0%, and mean BMI 30.5 ± 4.2 kg/m^2^). Mean energy intake was 2114 ± 649 kcal/day. DHEI scores were 17.0 (95%CI -6.8 to 41.0) points lower than HEI scores (55.9 ± 14.2% vs. 72.9 ± 10.7%, respectively; *P* < 0.001), suggesting there is no agreement (Bland-Altman method), and the Pearson correlation coefficient was 0.55 (*P* < 0.001). More patients were classified as having a low-quality diet by the DHEI than by the HEI (38.5% vs. 1.4%; *P* < 0.001). A higher proportion of patients (35.7%) with out-of-target total cholesterol levels had a low-quality diet evaluated by the DHEI (*P* = 0.03). We did not find associations between overall score of HEI and therapeutic targets.

**Conclusions:**

In its intended population of patients with type 2 diabetes, the DHEI seems to be a more rigorous tool to evaluate association between diet quality and changes in metabolic parameters.

**Electronic supplementary material:**

The online version of this article (10.1186/s12937-017-0296-8) contains supplementary material, which is available to authorized users.

## Background

Diabetes mellitus (DM) is a complex, chronic illness characterized by a heterogeneous set of metabolic disorders, including hyperglycemia and impaired carbohydrate, protein, and lipid metabolism, caused by abnormalities in insulin action and/or secretion [1]. It is consolidated as a serious public health problem at the national and international level, due to its high prevalence, marked morbidity and mortality, and the costs involved in its treatment, parallel to the increasing prevalence of obesity and sedentary lifestyle [1]. The adoption of healthy behaviors, especially changes in diet and physical activity, is an appropriate foundation for a DM self-management program of, and can serve as a basis for people with DM to achieve a healthy lifestyle [2].

Among nutritional recommendations for DM, the following dietary composition is recommended: 45 to 60% of daily calories from total carbohydrates (intake of not less than 130 g/day), 15 to 20% of daily calories from protein (or 0.8 to 1 g/kg/weight), and 25 to 35% of daily total calories from lipids. The latter are stratified as follows: <7% of daily calories from saturated fatty acids (SFA), up to 10% of daily calories from polyunsaturated fatty acids (PUFAs), 5 to 15% of daily calories from monounsaturated fatty acids (MUFAs), and daily cholesterol intake less than 300 mg [3]. The minimum dietary fiber recommendation is 14 g per 1000 kcal, with priority given to whole grains, vegetables, and fruits [3]. Under Brazilian guidelines, the recommended intake of vitamins and minerals is for individuals without diabetes [4], while sodium consumption should not exceed 2000 mg per day, which is equivalent to 5 g of cooking salt (i.e., one teaspoon of salt at most) [3].

Dietary indexes have been recommended to monitor adherence to nutritional recommendations among individuals and populations [5]. Several instruments are available to evaluate diet quality, among which the Healthy Eating Index (HEI) [6] stands out because it considers consumption both of food groups and of specific nutrients [6]. Recently, the Diabetes Healthy Eating Index (DHEI) [7] was developed, taking into account nutritional recommendations for this population, with the objective of evaluating diet quality and testing for possible associations with health outcomes in diabetics. Some studies have assessed the dietary quality of patients with DM, but none has used a dietary index that contemplates specific recommendations for this population [8, 9]. In this context, the objective of the present study was to compare the agreement of two dietary indexes (HEI and DHEI) to evaluate diet quality and possible association with therapeutic targets in patients with type 2 diabetes.

## Methods

### Study population

This was a cross-sectional study of outpatients with type 2 DM treated at Hospital de Clínicas de Porto Alegre (HCPA), a tertiary referral center in Southern Brazil, who were consecutively selected for the study “Quality of usual diet and health outcomes in patients with type 2 diabetes mellitus”. The diagnosis of type 2 DM was established as follows: disease onset after 30 years of age, no previous episodes of ketoacidosis or documented ketonuria, and treatment with insulin only ≥5 years after diagnosis [10]. Patients were included according to the following criteria: not having received dietary counseling from a nutritionist for at least 6 months prior to the study, age < 80 years, body mass index (BMI) <40 kg/m^2^, serum creatinine <2 mg/dL, normal thyroid function tests, and no kidney diseases (other than diabetic nephropathy), severe liver disease, decompensated heart failure, or any acute and/or consumptive illness. The study was conducted in accordance with the Declaration of Helsinki, and all procedures involving patients were approved by the Hospital de Clínicas de Porto Alegre Research Ethics Committee. Written informed consent was obtained from all patients.

### Clinical and anthropometric evaluation

Ethnicity information was obtained by self-report. Economic status was evaluated by a questionnaire designed according to the Brazilian reality [11]. The diagnosis of hypertension was established from readings obtained with an Omron model HEM-705CP blood pressure monitor [12]. The patient was considered hypertensive when mean systolic pressure was ≥140 mmHg or diastolic pressure ≥ 90 mmHg on at least two separate occasions, measured 1 min apart, or was receiving pharmacological treatment for hypertension, independently of blood pressure levels [13]. Diabetic nephropathy was assessed from spot urinary albumin excretion measurement; patients with values ≥14 mg/L were considered to have renal disease [14].

The anthropometric measurements used to assess nutritional status were weight (measured with patients wearing light clothing and barefoot), height, and waist circumference (measured at the midpoint between the lowest rib and the iliac crest) [15]. These measurements were obtained with an anthropometric scale and an inelastic fiberglass tape measure. BMI was calculated with the formula weight (kg)/height (m)^2^ [16] and its target value was set at <25 kg/m^2^ [1].

### Dietary assessment

Food intake information was obtained from a quantitative food frequency questionnaire (FFQ), previously validated in patients with type 2 DM [17], which contains 80 items with 10 food groups. A photo album was used to help patients select serving sizes. Reported intake was converted into daily consumption, and diet quality was evaluated using two dietary indexes: the HEI [6] and the DHEI [7]. Intake of individual portions was adjusted to a daily caloric intake of 1000 kcal. The Brazilian food composition table was used to evaluate the nutritional composition of the FFQ items [18].

The latest version of the HEI, used in this study, consists of 12 components [19]: nine food groups (“total fruit”, including 100% natural fruit juices; “whole fruit”, excluding juices and extracts; “total vegetables”; “greens and beans”; “whole grains”; “dairy”; “total protein foods”; “seafood and plant proteins”; “fatty acids”, distinguishing unsaturated and saturated) and three items referring to components to be consumed in moderation (“refined grains”, “sodium”, and “empty calories”, i.e., calories from solid fats, alcoholic beverages, and added sugar). The HEI is described in detail in Table 1. Individual scores range from 0 to 20 points, and the sum of the scores of each component yields a percent global score.

**Table 1:** Healthy Eating Index (HEI) components and criteria used for maximum score and zero score [19]

Components	Maximum Score	Maximum score criteria	Criteria for zero score
1. Total fruit (includes fruit juice)	5	≥ 0.8 cup per 1000 kcal	No fruit or juice
2. Whole fruit (includes all forms except juice)	5	≥ 0.4 cup per1000 kcal	No whole fruit
3. Total vegetables	5	≥ 1.1 cup per 1000 kcal	No vegetables
4. Greens and beans	5	≥ 0.2 cup per 1000 kcal	No dark green vegetables or legumes
5. Whole grains	10	≥ 40 g per 1000 kcal	No whole grains
6. Dairy	10	≥ 1.3 cup per1000 kcal	No dairy
7. Total protein foods	5	≥ 70 g per 1000 kcal	No protein foods
8. Seafood and plant proteins	5	≥ 20 g per 1000 kcal	No seafood or plant protein
9. Fatty acids	10	(PUFAs + MUFAs) / SFAs ≥ 2.5	(PUFAs + MUFAs) / SFAs ≤1.2
10. Refined grains	10	≤ 50 g per 1000 kcal	≥ 120 g per 1000 kcal
11. Sodium	10	≤ 1.1 g per 1000 kcal	≥ 2 g per 1000 kcal
12. Empty calories	20	≤ 19% of energy	≥ 50% of energy

*PUFAs* polyunsaturated fatty acids, *MUFAs* monounsaturated fatty acids, *SFAs* saturated fatty acids

The DHEI [7] consists of 10 components: six food groups (“fruit”, including fruit juices; “vegetables”; “carbohydrates and sources of fiber”; “meat and eggs”; “dairy and saturated fat”; “oils and fats”), three components referring to “percent daily calorie intake from lipids”, “dietary cholesterol”, and “*trans* fatty acids”; and a “diet variety” component. For the last component, each food was counted only when consumption was >50% the recommended intake in the corresponding food group. A score had been established for each component, the value of which is classified according to adherence to current national nutritional recommendations for DM, namely: “poor” (zero points), “fair” (one-half point), and “good” (one point) [7]. The sum of the scores of each component yields an overall score of diet quality, which it converted on a scale of zero to 100. The DHEI is described in detail in Table 2. In both indexes, the overall diet quality is classified as low (<51%), needing improvement (51–80%), or adequate (> 80 points) [19].

**Table 2:** Diabetes Healthy Eating Index (DHEI) components and criteria for adherence [7]

Components (daily intake)	Portion (kcal) [27]	Criteria for adherence with diabetes recommendations		
		Poor	Fair	Good
1. Diet variety: number of items	–	< 6	6–16	≥16
2. Fresh fruit (portions per 1000 kcal)	70	< 1.0	1.0–1.5	≥1 ½
3. Vegetables (portions per 1000 kcal)	15	< 1.0	1.0–1.5	≥1 ½
4. Carbohydrates and fiber sources (portions per 1000 kcal)	150	< 3	< 3 BUT at least 50% from fiber sources	≥3 AND at least 50% from fiber sources
5. Meat and eggs (portions per 1000 kcal)	190	>1.0	0.5–1.0	≤0.5
6. Dairy products (portions per 1000 kcal) AND saturated fatty acids (% of energy)	120	< 0.75 portion/day of dairy OR saturated fatty acids intake > 10.5% of energy	> 0.5 portion of dairy AND Saturated Fatty Acids < 7.0% of energy OR > 0.75 portion of dairy AND saturated fatty acids between 7.0 and 10.5% of energy	1.0–2.0 portions/day of dairy AND saturated fatty acids < 7% of energy
7. Oils, fats, and nuts (portions per 1000 kcal)	73	> 1.0	0.5–1.0	≤0.5
8. Total lipids (% of energy)	–	≥45%	30–45%	≤30%
9. Dietary cholesterol (mg/day)	–	≥450	300–450	≤300
10. *Trans-*unsaturated fatty acids (% of energy)	–	≥1.5%	1.0–1.5%	≤1.0%

Criteria for adherence were based on the Brazilian Society for Diabetes [3], Brazilian Dietary Guidelines [27], and original HEI [6] for classification of the diet variety component

### Laboratory measurements

Blood samples were obtained after a 12-h fast. Plasma glucose was determined using the glucose oxidase method (biodiagnostica Kit) [20]; glycated hemoglobin (HbA1c, reference range 4.7–6.0%), by high-precision chromatography in a Merck-Hitachi 9100 system [21]; and total cholesterol, high-density lipoprotein (HDL) cholesterol, and triglycerides (TG) by enzymatic colorimetric methods [22]. Low-density lipoprotein (LDL) cholesterol was calculated using the Friedewald eq. (LDL = total cholesterol – HDL – TG/5) [23]. Serum creatinine was measured by the Jaffé method [24]. All tests were performed at the Clinical Pathology Laboratory, Hospital de Clínicas de Porto Alegre.

### Statistical analysis

The distribution of variables was assessed using the Shapiro-Wilk test. Data were expressed as mean ± standard deviation or absolute and relative frequency (%), as indicated. Comparison of overall diet quality between the dietary indexes was done using the paired *t*-test. Analysis of concordance between the HEI and DHEI was performed using the Bland-Altman plot method, which evaluates the mean difference between two methods and considers the variability in these differences among individuals [25]. Poisson regression models were used to test for possible associations between individual component and overall diet quality of each of the dietary indexes with therapeutic targets (dependent variable). Analyses were adjusted to possible confounding variables selected according to clinical relevance. A chi-square test was used to evaluate the relationship between diet quality categories within each index and the achievement of therapeutic targets (outcomes). All data analyses were performed in PASW Statistics 18.0 (SPSS Inc., Chicago, IL), and the type I error rate was set at 5% (two-tailed).

Estimation of sample size was based on a pilot study previously performed in a subsample of 201 patients with type 2 DM, in which diet quality evaluated by the original HEI [6] was found to differ from DHEI scores (80.2 ± 11.7% vs. 61.7 ± 11.5%, respectively, *P* < 0.001). Thus, expecting a difference in diet quality of at least 10% among dietary indexes and considering a type I error of 5% and a type II error of 10%, 58 patients would be necessary.

## Results

A total of 148 patients with type 2 DM were consecutively included after the pilot study and were analyzed. Mean age was 63.2 ± 9.4 years; 73% were white, and 62.8% female. Mean A1c was 8.4 ± 2.0%, and mean BMI, 30.5 ± 4.2 kg/m^2^. Regarding dietary characteristics, the mean reported total calorie intake was 2114 ± 649 kcal/day. Table 3 describes demographic, clinical, lifestyle, and laboratory characteristics of the sample.

**Table 3:** Demographic, clinical, lifestyle and laboratory characteristics of patients with type 2 diabetes

Characteristic	
N	148
Age (years)	63.2 ± 9.4
Female	62.8%
White skin color	73.0%
Lower middle class	46.0%
Years of study	7.1 ± 3.7
Duration of diabetes (years)	10 [5–19]
Hypertension	
Systolic blood pressure (mmHg)	140.8 ± 21.9
Diastolic blood pressure (mmHg)	76.5 ± 11.5
Current smoking	6.8%
Micro- or macroalbuminuria	24.2%
BMI (kg/m^2^)	30.5 ± 4.2
BMI >30 kg/m^2^	56.1%
Waist circumference (cm)	
Males	105.5 ± 10.9
Females	101.9 ± 9.9
Diabetes treatment	
Diet alone	2.7%
Oral agents	43.2%
Insulin	6.1%
Insulin plus oral agents	48.0%
Hypolipidemic agents	68.2%
Fasting plasma glucose (mg/dL)	161.3 ± 71.9
A1c (%)	8.4 ± 2.0
A1c <7%	27.0%
Total cholesterol (mg/dL)	174.7 ± 40.2
Total cholesterol <200 mg/dL	53.4%
HDL cholesterol (mg/dL)	41.9 ± 10.0
Males	40.5 ± 8.9
Females	42.8 ± 10.6
LDL cholesterol (mg/dL)	100.6 ± 34.4
LDL cholesterol <100 mg/dL	50.7%
Triglycerides (mg/dL)	163.2 ± 88.2
Triglycerides <150 mg/dL	44.6%
Serum creatinine (mg/dL)	0.8 ± 0.2
Glomerular filtration rate (mL/min/1.73 m^2^)	83.7 ± 22.3
Glomerular filtration rate > 90 mL/min/1.73 m^2^	20.9%

Data presented as mean ± standard deviation, median [interquartile range] or n (%)

**Fig. 1:**
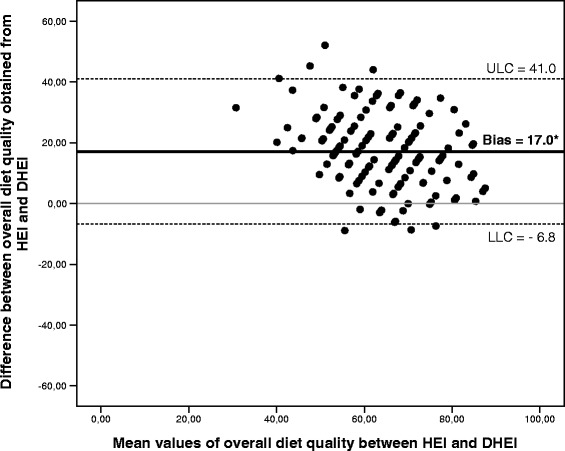
Bland-Altman plot between HEI and DHEI for determining overall diet quality of patients with type 2 diabetes (*n* = 148). The solid line represents the mean difference between the two instruments, and the dotted lines represent the minimum and maximum differences between HEI and DHEI, where LLC is the lower limit of concordance and ULC is the upper limit of concordance

Fig. 1 shows the Bland–Altman plots between the two dietary indexes. The mean difference (agreement range) observed between diet quality score evaluated by the HEI as compared to DHEI was 17.0 points (95%CI −6.8 to 41.0; *P* < 0.001).

**Fig. 2:**
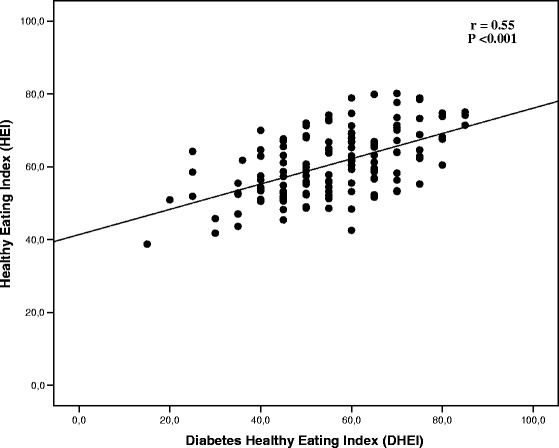
Pearson correlation coefficients (*r*) between HEI and DHEI scores in patients with type 2 diabetes (*n* = 148)

Pearson correlation between the overall diet quality assessed by the two indexes was calculated as *r* = 0.55 (*P* < 0.001), and is shown in Fig. 2. More patients were classified as having a low-quality diet by the DHEI than by the HEI (38.5% vs. 1.4%; *P* < 0.001).

Poisson regression models were also used to test for possible associations between overall diet quality of each of the dietary indexes and their individual components with therapeutic targets (dependent variable). Regarding the HEI, some individual components as total fruit, total protein foods, whole grains, sodium, empty calories and fatty acids were associated with therapeutic targets. Regarding the DHEI, other individual components as vegetables, meat and eggs, dietary cholesterol, and overall diet quality were associated with therapeutic targets (see Additional file 1).

A higher proportion of patients (35.7%) with out-of-target total cholesterol levels had a low-quality diet evaluated by the DHEI (*P* = 0.03). No association was found between overall HEI score and any of the therapeutic targets evaluated.

## Discussion

The overall diet quality score from the two dietary indices in this sample of 148 patients with type 2 DM was 72.9 ± 10.7% in the HEI and 55.9 ± 14.2% in the DHEI (*P* < 0.001). The mean score yielded by both instruments classified diet as “needing improvement”, similar to what was found in a recent systematic review of studies that evaluated diet quality in the Brazilian population [26]. Among the main results of the present study, we observed a moderate correlation between the two dietary indexes and a significant difference in the overall diet quality of approximately 17 points, as shown by the Bland-Altman plot method, suggesting that there is no agreement between the two instruments.

Thus, a greater propensity for rigidity of the DHEI is noted, possibly because it follows specific recommendations for the evaluated population. More patients were classified as having poor diet quality by the DHEI than by the HEI (38.5% vs. 1.4%, *P* < 0.001), and a higher proportion of patients (35.7%) with out-of-target total cholesterol levels had a low-quality diet evaluated by the DHEI (*P* = 0.03).

Associations between individual HEI consumption scores of components total fruit, total protein foods, whole grains, sodium, empty calories and fatty acids with therapeutic targets were observed (*P* < 0.05). Regarding the DHEI, some individual components as vegetables, meat and eggs, dietary cholesterol, and overall diet quality were associated with therapeutic targets (*P* < 0.05) after adjustment for confounders. However, a larger sample is needed to confirm these findings.

Recent studies evaluated the diet quality of patients with DM, but did not use a specific dietary index designed to take nutritional recommendations for this population into account [8, 9]. Regarding the association with health outcomes, only one cross-sectional study, conducted in a Korean population with type 2 DM [8], evaluated the association between glycemic control and diet quality as assessed by three dietary indexes: Diet Quality Index (DQI), alternative HEI, and Healthy Diet Indicator (HDI). The authors found an inverse correlation between DQI and HDI diet quality scores and A1c% (*r* = −0.21, *P* < 0.05, *r* = −0.28, *P* < 0.05, respectively), blood glucose (r = −0.21, *P* < 0.05, *r* = −0.23, *P* < 0.05, respectively), and postprandial glycaemia (*r* = −0.30, *P* < 0.05; *r* = −0.26, *P* < 0.05, respectively). Diet quality assessment with the alternative HEI was not associated with any of the outcomes evaluated.

Studies of diet quality in patients with DM are still scarce in the literature, especially when it comes to the use of instruments designed specifically for this population. In the present study, we used a new dietary index based on current nutritional recommendations for DM, with internal validity tested in a previous study [7]. Our objective was to evaluate its agreement with a widely used index [19] and to verify associations with therapeutic targets in type 2 DM.

The limitations of this study include the difficulty of comparing our results with those of other studies conducted in different populations of individuals with type 2 DM. In addition, use of these instruments is still beset by unanswered questions, such as which inferences can actually be drawn from their results and whether they are in fact able to evaluate the quality of eating habits as a whole without considering the impact of each food group has on overall diet quality. These methodological questions need to be evaluated carefully through repeated application in different populations if classification of diet quality is to become more reliable.

## Conclusion

The two instruments tested in this study (HEI and DHEI) had a moderate correlation, but no agreement. The DHEI seems to be a more rigorous instrument for evaluating the association between diet quality and achievement of metabolic targets in patients with type 2 DM.

## Additional files

Additional file 1: Poisson regression models between overall diet quality and individual components of dietary indexes with therapeutic targets (dependent variable) in patients with type 2 DM (*n* = 148). (DOCX 21 kb)

## Abbreviations

BMI: Body mass index
DHEI: Diabetes healthy eating index
DM: Diabetes mellitus
DQI: Diet quality index
FFQ: Food frequency questionnaire
HDI: Healthy diet indicator
HDL: High-density lipoprotein
HEI: Healthy eating index
LDL: Low-density lipoprotein
MUFAs: Monounsaturated fatty acids
PUFAs: Polyunsaturated fatty acids
SFA: Saturated fatty acids
TG: Triglycerides
