# Supplementary material for: Diet quality and therapeutic targets in patients with type 2 diabetes: evaluation of concordance between dietary indexes
Source: Nutr J. 2017 Nov 21;16:74. doi: 10.1186/s12937-017-0296-8 (PMC5696727; doi:10.1186/s12937-017-0296-8)
Supplement: Supplementary file 1 — Poisson regression models between overall diet quality and individual components of dietary indexes with therapeutic targets (dependent variable) in patients with type 2 DM (n = 148). (DOCX 21 kb) [file 12937_2017_296_MOESM1_ESM.docx]

**Additional File 1:** Poisson regression models between overall diet quality and individual components of dietary indexes with therapeutic targets (dependent variable) in patients with type 2 DM (n=148)

| Overall diet quality and individual components of dietary indexes | Therapeutic targets | Prevalence ratio (CI 95%) |
| --- | --- | --- |
| DIABETES HEALTHY EATING INDEX (DHEI)^7^ | | |
| Low diet quality (< 51%) | Total cholesterol <200mg/dL | 9.07 (2.50-32.70)** |
| Vegetables (1.0 to 1.5 portions per 1000 kcal) | Glycated hemoglobin <7% | 0.54 (0.30-0.98)* |
| Meat and eggs (0.5 to 1.0 portions per 1000 kcal) | LDL-cholesterol <100mg/dL | 3.02 (1.14-7.99)** |
| Dietary cholesterol (300 to 450 mg/day) | Body mass index <30kg/m² | 1.50 (1.01-2.24)* |
|  | LDL-cholesterol <100mg/dL | 2.21 (1.31-2.74)** |
| HEALTHY EATING INDEX (HEI)^19^ | | |
| Total fruit (cup per 1000kcal) | Glycated hemoglobin <7% | 0.86 (0.75-0.99)* |
| Total protein foods (g per 1000 kcal) | Body mass index <30kg/m² | 1.18 (1.02-1.36)* |
| Whole grains (g per 1000 kcal) | Fasting glucose 70-130mg/dL | 0.93 (0.87-0.99)* |
|  | Glycated hemoglobin <7% | 0.96 (0.92-0.99)* |
| Sodium (g per 1000 kcal) | Glycated hemoglobin <7% | 0.82 (0.70-0.95)** |
| Empty calories (% of energy) | Fasting glucose 70-130mg/dL | 0.81 (0.73-0.91)** |
|  | Glycated hemoglobin <7% | 0.90 (0.85-096)** |
|  | Total cholesterol <200mg/dL | 0.65 (0.49-0.88)** |
| Fatty acids (PUFAs+MUFAs)/SFAs | High waist circumference (cm) | 0.98 (0.96-0.99)* |

*P <0.05; **P <0.001

DHEI is expressed by categorical variables and HEI is expressed by continuous variables. Other dietary components were not associated with therapeutic targets.

All these analyses were adjusted to possible confounding variables selected according to clinical relevance model as gender, age, treatment and diabetes duration, body mass index, and physical activity.
